# Fetal Nasal Bone Length as a Novel Marker for Prediction of Adverse Perinatal Outcomes in the First-Trimester of Pregnancy

**DOI:** 10.4274/balkanmedj.2016.0133

**Published:** 2017-03-28

**Authors:** Mehmet Tunç Canda, Namık Demir, Orçun Sezer

**Affiliations:** 1 Clinic of Obstetrics and Gynaecology, Kent Hospital, İzmir, Turkey

**Keywords:** Nasal bone, perinatal outcome, fetal ultrasound, Turkish population

## Abstract

**Background:**

Adverse outcomes of pregnancy are a challenging health-care problem. Prediction of adverse pregnancy outcomes is important to prevent the morbidities of the foetus and the mother.

**Aims:**

To study the clinical interest of fetal nasal bone length in predicting adverse pregnancy outcomes in the first trimester of pregnancy.

**Study Design:**

A population-based retrospective cohort study.

**Methods:**

Data from 868 women with first-trimester fetal nasal bone length and birth records available were enrolled. Fetal nasal bone length percentiles were determined and evaluated for their ability to predict adverse pregnancy outcomes such as preterm birth, preterm labour, preterm premature rupture of membranes, early preterm birth, gestational diabetes mellitus, gestational hypertension-preeclampsia, small-for-gestational age foetuses, macrosomia, oligohydramnios, polyhydramnios and fetal distress.

**Results:**

Fetal nasal bone length >95^th^ percentile was significantly associated with preterm labor and preterm premature rupture of membranes (p=0.02, accuracy 0.91 and p=0.001, accuracy 0.94, respectively), whereas nasal bone length >99^th^ percentile was significantly associated with preterm labor and oligohydramnios (p=0.006, accuracy 0.95 and p=0.014, accuracy 0.97).

**Conclusion:**

Fetal nasal bone length at high percentiles in the first trimester of pregnancy may aid in the prediction of adverse outcomes such as preterm labour, preterm premature rupture of membranes and oligohydramnios.

Efforts to predict whether a pregnancy will be normal or have an adverse outcome have long been a part of obstetric practice. Previous studies have shown that low maternal serum pregnancy-associated plasma protein-A (PAPP-A) or free β-human chorionic gonadotrophin (fβ-hCG) in the first trimester could predict subsequent adverse perinatal outcomes (1,2). Anatomic investigation of the foetus in the first trimester of pregnancy has been emphasized because it yields the early detection of many conditions (3). Imaging techniques like the nuchal translucency thickness measurement have been crucial in the improvement of screening tests, both for chromosomal abnormalities and for adverse outcomes (4,5).

There is an ongoing effort to find new markers for predicting adverse outcomes in the first trimester of pregnancy. Previously, the role of nasal bone (NB) length (NBL) in screening for genetic defects was assessed (6,7). Furthermore, the possible relationship between fetal NBL and adverse perinatal outcomes is of interest.

The purpose of the present study was to determine the ability of first-trimester fetal NBL to predict adverse perinatal outcomes in women with singleton pregnancies who delivered healthy foetuses without apparent structural malformations.

## MATERIALS AND METHODS

### Study population

This study was performed after institutional review board approval and informed consent had been obtained. A retrospective chart analysis of 868 women who had first-trimester screening with fetal NBL measurements between their 11 and 13^+6^ gestational weeks was included. All cases had the full data of the prenatal visits and delivered in our institution. The study investigated the time period between January 2007 and December 2012. Gestational age of each case was determined according to both the last menstrual period and ultrasonography. If a discrepancy of more than one week was detected between these two parameters then these cases were excluded from the study. Pregnancies with an unknown or suspicious last menstrual period, those achieved by any infertility treatment, those with detected aneuploidy and/or a fetal anomaly, mothers with any chronic diseases (e.g. diabetes type 1 or 2, hypertension or rheumatic diseases), pregnancies with a complicated maternal obstetric history (previous pre-eclampsia, preterm delivery, gestational diabetes or stillbirth), multiple gestations including vanishing twins and pregnancies that underwent cervical cerclage were excluded. Additionally, women with a known history of alcohol, drug or tobacco abuse were not included in the study. The pregnancies with absent fetal NB were also excluded.

### First-trimester additional screening test parameters

**Figure 1:**
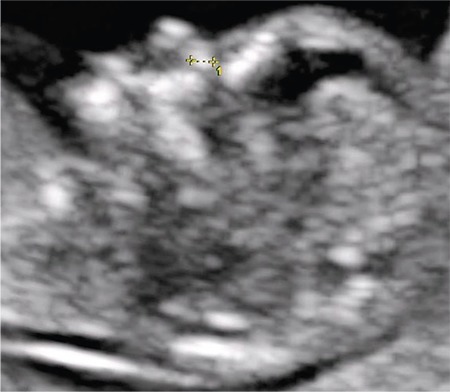
Foetal nasal bone length measurement. Note that the calipers are in the out-to-out position.

All pregnant women who attended the outpatient obstetric clinic were counselled about aneuploidy screening and were offered routine first-trimester screening. The standard screening was performed between 11 and 13^+6^ weeks of gestation (77-97 gestational days) and a crown-rump length (CRL) between 45 and 84 mm according to the guidelines outlined by the Fetal Medicine Foundation (www.fetalmedicine.com). Fetal NBL was measured by three sonographers, who had at least 2 years’ experience in these measurements, and standardized with audit and feedback. All ultrasound scans were performed with a Voluson^®^ E8 Expert (GE Healthcare, Wisconsin, USA) ultrasound machine using a wideband convex volume transducer transabdominal probe operating at a frequency of 2-8 MHz. For fetal NB measurement, a mid-sagittal view of fetal face in the supine position was obtained transabdominally with the transducer perpendicular to the fetal NB. The image on the screen included only the fetal head, neck and upper thorax. The increment in the distance between calipers for the magnification of image was set to 0.1 mm. The presence of the NB was determined by gently tilting the transducer from one side to another. This manoeuvre helped to identify the nasal tip and the overlying skin separately from the NB as three distinct lines. The one at the top symbolized the skin, the one at the bottom symbolized the NB and the last one continuous with the skin symbolized the nasal tip. An insonation angle of approximately 45º was used to make sure that the fetal NB was truly present. If the NB was more echogenic and thicker than the overlying skin, it was assumed to be present. If the NB was either the same or less echogenic or it was not visualized clearly then it was assumed to be absent (6,7,8,9). The fetal NBL was measured by placing the calipers in an out-to-out position (Figure 1).

### Adverse pregnancy outcomes

The pregnancy outcomes were obtained from a computerized perinatal database. The adverse perinatal outcomes were as follows: preterm birth, which was defined as any spontaneous delivery before 37 completed gestational weeks (<259 days); preterm labour, which was defined as labour (regular uterine contractions resulting in changes in the cervix including effacement and dilatation) before 37 completed gestational weeks (<259 days); and preterm premature rupture of membranes (PPROM), which was defined as rupture of the membranes before the onset of labour occurring before 37 completed gestational weeks (<259 days). Early preterm birth was defined as any spontaneous delivery before 34 completed gestational weeks (<238 days). Gestational diabetes mellitus (GDM) was diagnosed when patients had two or more positive values in a 3-hour 100 g oral glucose tolerance test and a previous high glucose challenge test. Gestational hypertension-pre-eclampsia Gestational hypertension was defined as blood pressure elevation after 20 weeks of gestation in the absence of proteinuria (at least 300 mg of protein in a 24-hour urine collection or 1+ or greater protein in a urine dipstick test) or any of the severe features of pre-eclampsia. Pre-eclampsia was defined as blood pressure elevation after 20 weeks of gestation with proteinuria or any of the severe features of pre-eclampsia. Small-for-gestational age (SGA) was defined as a birth weight below the 10^th^ percentile for the reference population (10). Fetal distress was diagnosed when non-reassuring patterns were detected on the fetal cardiotocograph during labour. Oligohydramnios was defined as an amniotic fluid index ≤5 cm. Polyhydramnios was defined as an amniotic fluid index ≥25 cm. Macrosomia was defined as a birth weight >4000 g. The adverse perinatal outcome group consisted of patients with one or more of the above conditions.

### Statistical analysis

Data were analysed with the SPSS software (SPSS version 16, SPSS Inc.; Chicago, IL, USA). The percentiles of fetal NBL were determined and evaluated for their ability to predict adverse perinatal outcomes. Medians were compared using the Mann-Whitney U test if the parameters were non-normally disturbed. Categorical variables were analysed using Pearson’s chi-square or Fisher’s exact tests. Linear correlations were analysed using Spearman’s correlation analysis. Statistical significance was defined as p<0.05. A sample size of 868 achieves 94% of the experimental power needed to detect an effect size of 0.1187 between preterm labour and fetal NBL >95^th^ percentile using a 1 degree of freedom chi-square test with a significance level (alpha) of 0.05. A regression formula for NBL was measured to identify the growth rate of NB according to CRL.

## RESULTS

There were 236 cases with adverse outcomes and 632 uneventful pregnancies. The adverse perinatal outcome group consisted of 79 cases of preterm birth, 45 cases of preterm labour, 25 cases of PPROM, 20 cases of early preterm birth, 94 cases of GDM, 12 cases of gestational hypertension-pre-eclampsia, 52 cases of SGA foetuses, 12 cases of fetal distress, 21 cases of oligohydramnios, 5 cases of polyhydramnios and 25 cases of macrosomia.

**Figure 2:**
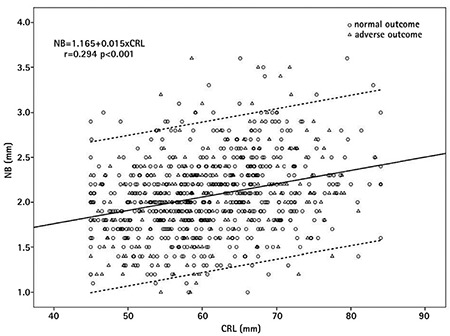
The length of the nasal bone (NB) in normal and adverse outcome foetuses in relation to the crown-rump length. ᴼ=normal outcome; Δ=adverse outcome foetuses. The solid line indicates the median, and the dotted lines are the 5^th^ and 95^th^ percentiles of the NB length.

Maternal age and weight were significantly higher in the adverse perinatal outcome group than the normal outcome group (p<0.001 and p=0.007, respectively). The NBL and CRL in the adverse perinatal outcome group and the normal outcome group were almost similar (p=0.673). Comparison of general characteristics of the adverse and normal outcome groups are summarized in Table 1. For the normal foetuses with measurable NBL, a regression line was calculated (Figure 2). The regression line demonstrated a significant positive slope with increasing CRL [NB=1.165 + 0.015X (CRL)] (r=0.294, p<0.001). While the CRL increased (from 45 to 84 mm), the NBL indicated a 31.5% increase (an increase from 1.84 to 2.42 mm).

The median (25-75 percentiles) for fetal NB was 2 mm (1.8-2.3 mm). The calculated 1^st^, 5^th^, 95^th^ and 99th percentiles for fetal NBL were 1.16 mm (0.58 MoM), 1.34 mm (0.67 MoM), 2.85 mm (1.42 MoM) and 3.33 mm (1.66 MoM), respectively. The mean NBL in female (n=414) and male (n=454) gender were almost equal (2.0693±0.438 and 2.0632±0.452, respectively; p=0.84).

Preterm labour was significantly associated with fetal NBL >95^th^ percentile and fetal NBL >99^th^ percentile (p=0.02, accuracy 0.91 and p=0.006, accuracy 0.95, respectively). In addition, PPROM was significantly associated with fetal NBL >95^th^ percentile (p=0.001, accuracy 0.94). Oligohydramnios was significantly associated with fetal NBL >99^th^ percentile (p=0.014, accuracy 0.97). No association was found for adverse perinatal outcomes such as preterm birth, early preterm birth, GDM, gestational hypertension-pre-eclampsia, fetal distress, SGA and macrosomia. Additionally we found some associations between PAPP-A, fβ-hCG and adverse outcomes such as: PAPP-A <1^st^ percentile and fβ-hCG >95^th^ percentile is associated with adverse pregnancy outcome (p=0.038 and p=0.034, respectively); fβ-hCG >95^th^ percentile is associated with fetal macrosomia (p<0.01). PAPP-A <1^st^ percentile; and PAPP-A <5^th^ percentile is associated with SGA foetuses (p<0.001 and p=0.003, respectively).

## DISCUSSION

A NBL in the high percentiles was found to be strongly associated with preterm labour, PPROM and oligohydramnios in the present study. Previously, first-trimester screening test parameters including nuchal translucency, serum fβ-hCG, PAPP-A and ductus venosus were investigated for their associations with adverse perinatal outcomes (1,2,4,5,11). NB is one of the additional parameters of the first-trimester screening test and usually its absence or hypoplasia is found to be associated with chromosomal abnormalities (6,12). However, the possible association of NBL and the adverse perinatal outcome has not yet been assessed.

The absence of the NB was observed in 73% of Down syndrome cases and 0.5% of euploid foetuses (13). Additionally, the association between increased NBL and adverse perinatal outcomes is noteworthy. Previously, Sonek et al. (14) speculated that NBL may inversely correlate with the risk of aneuploidy. According to the results of the present study, we may speculate that NBL at high percentiles may be directly correlated with some adverse perinatal outcomes.

We could not find any reliable and rational mechanism that reveals the association between NBL and preterm labour, PPROM and oligohydramnios from the physiologic point of view through a PubMed search.

Low sample size may be an important limitation of our study; however, we selected patients using strict criteria. Our data are notably homogeneous in terms of ethnicity, especially compared to multicentre studies; this is also true for the few investigators with high compatibility. Additional affirmative strengths of the study include the 100% follow-up of all patients and an accurate account of adverse outcomes. In addition, different from most published studies, we investigated many adverse outcomes of pregnancy with distinctive aetiologies. Another concern that may arise is the feasibility of the fetal NBL measurements that may vary between operators. However, after a sufficient period of training, they may be easily incorporated to the first-trimester screening test parameters. Moreover, fetal NBL measurements in our study were comparable to previous studies from Turkey (7,15,16) and from other ethnicities (6,17).

Briefly, a challenging observation was made in the present study, which is the first-time report of an association between increased NBL and preterm labour, PPROM and oligohydramnios. This association has not been addressed in the literature previously and whether this association is a coincidence needs to be confirmed in studies that include larger numbers of patients. The possible reliability of these associations should be confirmed with other studies related in this field before incorporating them into clinical practice. The ability of fetal NBL to detect adverse outcomes may be of interest to others in this field planning extensive research. Fetal NBL may serve as a potential candidate for screening for adverse perinatal outcomes.

## Figures and Tables

**Table 1:**
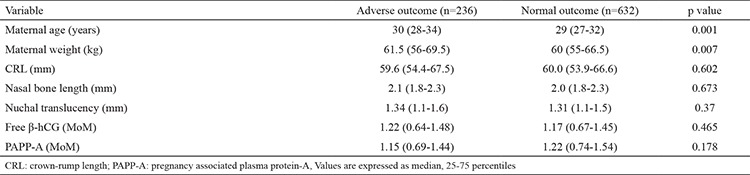
Comparison of various parameters between adverse and normal pregnancy outcomes
